# Bias of Calf Sex on Milk Yield and Fat Yield in Holstein Crossbreed Cows

**DOI:** 10.3390/ani11092536

**Published:** 2021-08-29

**Authors:** Radica Djedović, Dragan Stanojević, Vladan Bogdanović, Dušica Ostojić Andrić, Ljiljana Samolovac, Tamara Stamenić

**Affiliations:** 1 Department of Animal Science, Faculty of Agriculture, University of Belgrade, Nemanjina 6, 11080 Belgrade, Serbia; stanojevic@agrif.bg.ac.rs (D.S.); vlbogd@agrif.bg.ac.rs (V.B.); 2 Institute for Animal Husbandry, 11080 Belgrade, Serbia; andricdusica.iah@gmail.com (D.O.A.); ljiljanasamolovac@gmail.com (L.S.); tstamenic169@gmail.com (T.S.)

**Keywords:** Trivers–Willard hypothesis, sex, calf, dairy cattle, milk traits, maternal investment

## Abstract

**Simple Summary:**

Cattle comprise a species of a domestic animal that is primarily bred for milk production. The birth of a calf is the initiator for the lactation period and the sex of the calf can affect milk yield. Additionally, a calf from a new pregnancy can affect the lactation from the previous calving, since the mother gets pregnant and remains pregnant during most of the lactation, usually at lactation peak. Therefore, the aim of this paper was to further investigate the possibilities of sexually biased milk production of Holstein and Holstein crossbreed cows using data from the Republic of Serbia. We also wanted to test the Trivers–Willard (TW) hypothesis that natural selection favors unequal parental investment between daughters and sons under certain maternal conditions. At the same time, this hypothesis assumes that mothers in good health and condition invest more in sons, while mothers in a poor condition invest more in daughters. The obtained results deviate from the view of the TW hypothesis because it was found that milk and fat yields in the first two lactations were the highest in cows that had a female calf and were then pregnant with a second female calf while the lactation from the previous calving was still in progress. We were the first in the world to investigate the effect of the sex of calves at first and second calving on milk yield and fat yield in the first and second standard lactation, depending on milk production levels on farms.

**Abstract:**

In order to examine the biased milk production depending on the sex of calves, data on calving and milk yield characteristics of 15,181 Holstein type cows in PK Belgrade, Serbia were analyzed. A total of 30,362 lactations that were realized in the period from 1985 to 2017 were analyzed. Data were prepared and analyzed using the SAS software package (SAS Institute Inc. Software License 9.3, 2012). The expression and variability of investigated traits were determined using the PROC MEANS procedure, while the effect of individual factors on milk yield traits was analyzed using the PROC GLM procedure. Obtained results deviate from the views of the Trivers–Willard (TW) hypothesis. The results indicate that mothers invest more in female offspring by producing a higher milk and fat yield in the first and second lactation compared to male offspring. This is especially emphasized under better environmental conditions. The highest milk yield (7788 kg) and fat yield (271 kg) in the second lactation were achieved in the combination with two consecutive female calves in the group of higher-than-average milk production farms, and lowest in the combination of two consecutive male calves (6783 kg for the MY and 243 kg for the FY), respectively.

## 1. Introduction

Cattle breeders improve their genetic basis via artificial selection by favoring individuals with higher production capacities and excluding those with lower yields, thus over generations accumulating desirable genes that influence the improvement of the most important traits, such as milk production and fertility. Systematic record keeping and production control for a large number of animals in the population have enabled dairy cattle to become model organisms for examining mechanisms of milk synthesis and the effect of sex of the offspring on milk yield traits [1]. Studies have shown that the growth rate of a suckling male calf is higher than that of female calves. Therefore, it would be expected for cows to have higher milk production or higher milk quality and energy while nursing a male [2].

For several decades already, researchers have been asking the question and searching for the answer to the question of if mammalian mothers can adaptively control and invest in the sex of their offspring? According to the TW hypothesis [3], mothers in a good condition invest more in sons because they can provide more offspring, and achieve higher reproductive success through preferred investment over daughters, while mothers in the poor condition (among other things, reared under poor conditions and, for example, measured by weight) invest more in daughters because it is believed that daughters of poorer constitution will give birth to more offspring than weaker sons. In addition to results obtained by the previous study [3], this theory has also been tested on various mammalian species by other researchers [1,4,5,6,7].

According to the TW hypothesis, it has been established that the sex of offspring can also have an effect on the energy content of milk, with milk produced for male offspring being denser (containing more fat) in well-fed mothers [8].

The link between calf sex and milk yield traits has been the subject of various studies, but results of the analyses published so far are very different. Beavers and Doormaal [9] found in their research that the overall increase in milk yield in cows whose first two calves were female (HH) compared to cows with male calves (BB) was 76 kg (0.4%). This increase in milk yield is much lower than the value published in the earlier study by Hinde and colleagues [1] who, with the same calving order combination, found 2.7% or 445 kg in favor of female calves. Data from Iran [10] also indicate slightly higher milk production in mother cows that calved female calves in four consecutive lactations. Similar research in New Zealand [10,11] established a small positive effect of female calves on the total milk yield of Holstein Friesian and Jersey cows. In contrast, various studies [12,13,14] found that cows have a higher milk yield in the case of a male rather than a female calf.

If sexed semen is used, the chances of obtaining a female calf increase from about 50 to 85 percent or higher. Studies [15,16] on the impact of sex on milk production are very important because they can increase production results, especially if sexed semen is used to inseminate heifers.

Because published results of research on the possible impact of the sex of calves on milk production are very inconsistent, this study aimed to further explore the possibilities of sex-biased milk production of Holstein type dairy cattle depending on the sex of calves. In addition to monitoring the bias of calf sex on milk yield and milk fat yield in the first two lactations of Holstein crossbreed cows, we also tested the Trivers–Willard hypothesis regarding unequal parental investment between daughters and sons in different conditions in which mothers produce milk. The body weight of an animal is influenced by its age, genotype and the environmental conditions under which it is raised. Environmental conditions, which are conducive to large size, also contribute to high levels of production. Thus, large cows may give more milk, not only because they are large but because they are maintained under better conditions than smaller cows [17]. Additionally, reports [18] provide evidence that the magnitude and pattern of BW changes in the first weeks of lactation are of utmost importance for the subsequent reproductive performance of cows. Therefore, as a criterion for conditions in which mothers produce milk, we observed not only the milk yield on the farm, but also the body weight of the mothers themselves.

## 2. Materials and Methods

### 2.1. Materials

Data on milk yield, fat content, fat yield, duration of lactation, as well as the sex of calves at birth were gathered by the service that keeps records and controls cattle productivity at PK Beograd. The analysis included data for the period from 1985 to 2017. The first set of criteria for forming a data set for analysis for this paper was that each animal had at least the first two concluded lactations, as well as data on the sex of calves born. Records for lactations that began and ended with the birth of twins and during which abortions and stillborn calves were recorded were excluded from the original database. Since milk yield decreases after a difficult calving (3.8% in this dataset), all cows that had difficult calvings were excluded from the analysis, i.e., only calvings without assistance and with low level assistance (milder pulling) were analyzed. Lactation duration was adjusted to a standard length of 305 days. In addition, lactations shorter than 210 days and longer than 480 days were excluded. Lactation duration was limited to up to 480 days in order to avoid large variations in milk yield characteristics as a consequence of the duration of the service period. Additionally, we believe that this restriction avoids the impact of prolonged lactation on production in the next lactation.

Neither sexed semen nor recombinant bovine somatotropin were used for the cows included in the analysis since the use of this hormone is not allowed in Serbia. Set criteria led to the formation of a data set of 30,362 lactations, i.e., data were recorded for a total of 15,181 black and white cows producing on 7 PK Beograd farms and originating from a total of 382 bulls of the Holstein Friesian breed. Until September 2018, the PKB Corporation owned about 30,000 hectares of agricultural land and had 9000 dairy cows. Since 2018, the company has been managed by Al Dahra Serbia (coordinates: 44°59′59.6″ N, 20°24′17.2″ E).

Cows were reared on farms with a tie-stall housing system, in standardized facilities and fed a TMR meal that was uniform throughout the year. The use of TMR meals in the cow’s diet allows animals to consume a uniform mixture of appropriate amounts of all nutrients (bulky, concentrated, mineral and vitamin supplements) that make up an adequate meal. The quality and composition of meals within farms differ according to production groups: freshly calved cows in the calving unit, cows during the first 60 days of lactation, and cows in the middle of lactation—according to the level of milk yield and dry cows. Nutrition technology on all farms was the same. Differences that may have existed between farms were those regarding the chemical composition of bulk nutrients, depending on soil quality and food preparation technology (basically the technology of preparation was the same, and any differences existed only in relation to workers, application of adequate mechanization, time of preparation of bulk nutrients, biological phase of plants at mowing, etc.).

The main components of the ration consisted of corn silage, alfalfa hay, canola meal, wheat bran and feed additives and, later on, following their first calving, a total mixed ration was introduced.

Each animal included in the analysis had the following data: identification number, origin two generations back, lactation number, calving date, calf sex, as well as following production-related information: duration of lactation, total milk yield and a standard 305-day lactation, fat content and fat yield.

The bias of the sex of calves on milk yield (MY) and fat yield (FY) was studied after calving and the completion of the first two lactations of mothers and did not include mechanisms that affect the endocrine control of mammogenesis and lactogenesis.

In order to examine the first part of the TW hypothesis regarding the question of whether mothers invest differently in the sex of their offspring by producing more milk and higher fat content and yield for the development of one compared to the other sex (heifer—H; bull—B), all mothers analyzed in this study were divided into 4 categories based on different possible combinations of the sex of calves after the first two consecutive calvings. Data for cows that calved a female calf twice in a row were labeled “HH”, those with two consecutive male calves with “BB”. Other data were grouped as “HB” or “BH” according to the sex of the first and second calved calves by Hinde et al. [1].

The second part of the TW hypothesis assumes that well-nourished mothers invest more in male offspring, as strong sons will more likely leave more offspring, whereas even weaker daughters will produce more progeny than weak sons [3].

The data used in this study were selected based on the criteria listed in Table 1.

To test this part of the TW hypothesis, farms were divided into two groups (above and below average milk production farms) as can be seen in Table 2. The criteria, based on which farms were divided into these groups. were the average milk yield of cows in the first two standard lactations (14,337 kg) and average body weight (BW). On farms with above-average milk production, it was noticed that the BW of the examined animals was higher than the average, which was >520 kg for first-calvers and >670 kg for cows, as opposed to animals on below-average farms where body weight was below the stated averages (Table 1).

On all observed farms the same bulls were used for breeding, meaning that there were no differences in genetic potential and that the differences in milk yield of these two groups of farms resulted from different management and other non-genetic environmental factors. The sex ratio in the examined period for male and female calves was 51.5:48.5. The average insemination index for heifers was 1.8 and for older cows 3.5 (in extreme cases even up to 7 during summer months). At all times, insemination was performed on each farm with 6 bulls, of which 2 for heifers, 2 for cows, one as a spare in case of kinship, and additionally 1 young bull in testing. All bulls (382 in total) had over 5 daughters on each farm. The number of daughters per sire ranged from 17 to 150. The genetic potential of bulls, i.e., BV (breeding value) for analyzed milk yield traits is known.

### 2.2. Statistical Analyses and Models

(1) Two models were used to analyze the existence of sex bias on milk yield and fat yield in a standard lactation after two consecutive calvings. The first model that was tested, in addition to the fixed effects of the farm, year of calving, season of calving, calving number (parity), the share of Holstein Friesian genes, the effect of milk yield levels on farms, the effect of a male or female calf (B, H), also contained the interaction between sex and lactation order.

The mentioned model was as follows:

Y_ijklmsn_= µ + F_i_ + G_j_+ S_k_ + L_l_ + H_m_+ K_s_ + R_n_ + (R × L)_nl_ + e_ijklmsn_

where:

Y_ijklmsn_—phenotypic expression of the investigated trait;
µ—general average of the population;
F_i_—fixed effect of i-th farm (i = 1–7);
G_j_—fixed effect of j-th year of calving (j = 1985–2017);
S_k_—fixed effect of k-th season of calving (k = 1–4; seasons: winter (December, January and February), spring (December, January and February), summer (June, July and August), and autumn (September, October and November));
L_l_—fixed effect of l-th lactation (l = 1–2);
H_m_—fixed effect of the group according to the share of genes of the Holstein Friesian breed, 1st group—animals with 0 to 50% share of genes of the Holstein Friesian breed; 2nd group—animals with 51 to 75% share of genes of the Holstein Friesian breed; 3rd group—animals with 76 to 87.5% share of genes of the Holstein Friesian breed; 4th group—animals with 88 to 93.75 share of genes of the Holstein Friesian breed; 5th group—animals with more than 93.5% share of genes of Holstein Friesian breed);
K_s_—fixed effect of the level of milk yield on farms (above and below average farms; (s = 1–2);
R_n_—fixed effect of sex of the calf (*n* = H,B; heifer-H; bull-B);
(RxL)_nl_—interaction between sex and lactation order; (nl = 1–4);
e_ijklmsn_—random error.

(2) The effect of sex of calves during the first and second pregnancy in the first and second lactation on investigated milk yield traits was examined by another model in which, in addition to the above fixed factors, all four combinations of female and male calves (HH, HB, BH, BB) were included, as well as the interaction between combinations of sex of calves (sex class) and milk yield levels on farms with above and below average milk yield (*n* = 1–8)

where:

R_n_—effect of calf sex class (HH, HB, BH, BB);
(R × K)_ns_—interaction between calf sex class and level of milk yield on farms (above and below average farms);
e_ijklmsn_—random error of observation.

Statistical analysis was performed using the GLM procedure within the SAS software package (Version 9.3.; SAS Institute, Cary, NC, USA). Analysis of variance was used to assess the significance of the effect of factors on investigated traits, while the significance of differences was estimated using the *t*-test [SAS, 2013]. Analysis of variance ANOVA was also used to determine the preferred effect of sex in 4 combinations (HH, HB, BH, BB) on milk yield traits.

## 3. Results

The results in Table 3 indicate that Holstein type cows in both the first and second lactation periods produced more milk and more fat after the birth of daughters compared to sons. Cows that calved daughters after the first calving produced 80 kg more milk and 2 kg more fat in the first standard lactation of 305 days than those that calved sons (6777 kg of milk and 242 kg of fat versus 6697 kg of milk and 240 kg of fat). In the second lactation, yields of milk and fat were also higher after daughters were born.

The difference in the favor of female calves for the first two lactations was 84 kg of milk and 3 kg of fat. Statistically significant differences for MY in the first and second lactation were at the level of *p* ≤ 0.01, while for FY in the first lactation, they were significant at the level of *p* ≤ 0.05. A statistically significant difference in FY applied only to first calved cows, while in the second calving, no significance was found.

Using analysis of variance, it was found that the investigated traits (MY and FY) were significantly affected by almost all estimated factors, except for calving season and combination of sex of calves at first and second calving on FY (Table 4).

**Figure 1:**
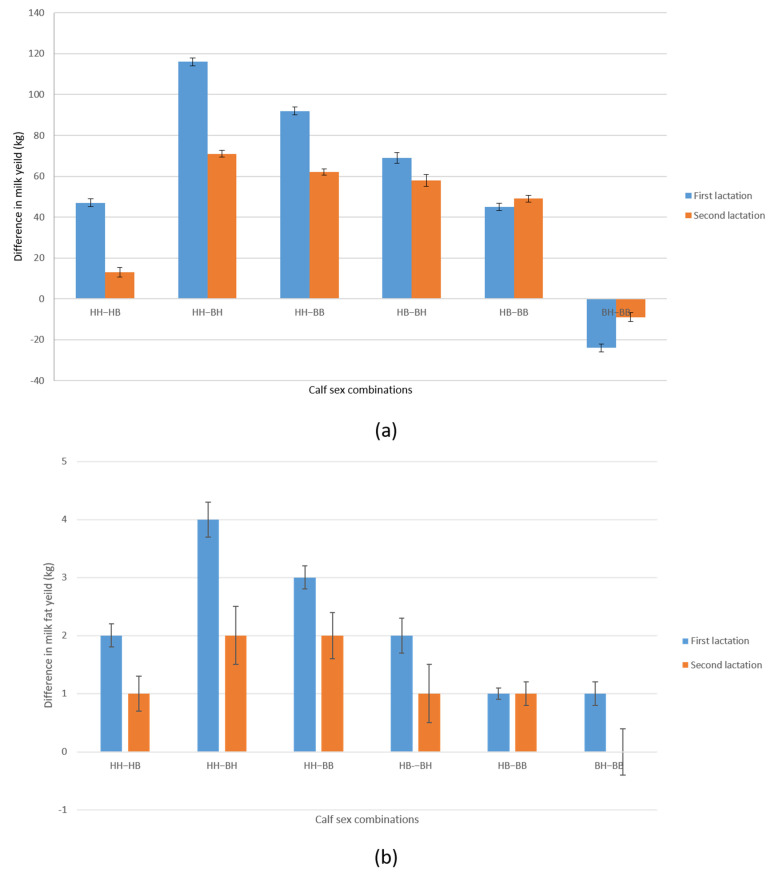
Differences in milk yield ±SE (part (**a**)) and milk fat yield ± SE (part (**b**)) in first and second standard lactation (305 days) between possible combinations of calf sexes (H = heifer; B = bull) by birth order (HH-HB; HH-BH; HH-BB; HB-BH, BH-BB). Analysis of 15,181 Holstein crossbreed cows produced on farms in the Republic of Serbia in the period from 1985 to 2017 showed that the largest difference in MY in first and second lactation is observed between combinations (HH-BH and HH-BB). As it is known that milk yield and milk fat yield are in high genetic correlation and in milk fat yield in first and second lactation, the largest differences between the same combinations of calf sexes in the order of birth (HH-BH and HH-BB) were recorded. The determined differences between the observed sex combinations were statistically significant (*p* < 0.01); SE—standard error.

As shown in Table 5, MY in the first lactation was highest in cows that calved a female calf and were then pregnant during the first lactation with a second female calf. Thus, cows that calved two female calves (HH = 6801 kg; 243 kg) produced 47 kg of milk and 2 kg of fat more in the first lactation (305 days) than cows that first calved a female calf and later became pregnant and carried a male calf (HB). An even greater difference (69 kg) in MY in the first lactation was observed between cows from categories HB (6754 kg) and BH (6685 kg). However, the greatest bias and difference in MY and FY in the first lactation (92 kg and 3 kg), respectively (Figure 1), was observed between cows that calved the first two female (HH) and two male calves (BB = 6709 kg; 240 kg). A similar pattern in MY and FY was also observed in the second lactation, for the difference between categories HH (7634 kg; 270 kg) and BB (7572; 268 kg), in this case amounting to 62 kg and 2 kg, respectively. The cumulative gain, i.e., the difference in MY between two consecutive calvings with two female calves (HH) and two male calves (BB) in the first two lactations was 154 kg.

Differences between combinations of calf sex within lactations and between lactations within calf sex combinations were statistically highly significant (*p* < 0.01), i.e., statistically significant at the level *p* < 0.05.

The effect of the observed four combinations of calf sexes at first and second calving on milk yield and fat yield depending on production levels on farms (Table 6) showed that the highest MY and FY in the first lactation were achieved on farms with above average milk production in the category of two of first-calved female calves (HH = 6962 kg; 246 kg), respectively, while the lowest MY and FY were achieved in the group of below average milk production farms when combined with two first-calved male calves (BB = 6061 kg; 220 kg), which was also a difference of as much as 901 kg milk and 26 kg of fat. In the second lactation, the highest MY and FY was achieved in the HH combination (7788 kg; 271 kg) in the group of above average farms, and the lowest in the BB combination (6783 kg; 243 kg), respectively, with an even greater difference between calf sexes of 1005 kg of milk and 23 kg of fat.

Differences between combinations of sex of calves within lactations and between lactations, as well as within and between farms were statistically significant (*p* ≤ 0.01).

## 4. Discussion

As mentioned in various studies [5,7,19,20,21], milk synthesis in different mammalian species is probably the most energy-valuable component of a mother’s investment in offspring. The above authors suggest that the sex of the calf in utero affects the endocrine control of mammogenesis. It is generally accepted that prolactin and placental lactogens play an important role in mammogenesis and lactogenesis [22,23,24,25,26]. Prolactin, growth hormone, and placental lactogens form a group of structurally linked hormones, which probably evolved from a peptide that belonged to a common ancestor. Prolactin and the growth hormone are present in all mammals, while biological activity associated with placental lactogen has been found only in some mammalian species. Placental lactogen activity has been detected in primates, some rodents, llamas, giraffes, several species of deer, antelope, gazelle, mouflon, buffalo, some breeds of sheep, goats, and cows. Milk secretion is a complex process influenced by a wide range of factors, including diet and genetic potential, but the role of the fetal sex of the calf is not negligible [1]. It has been proven that prolactin and growth hormone play a key role in stimulating mammary gland development, its differentiation, and milk secretion function by adapting to maternal metabolism and the stages of pregnancy and lactation [22].

We found that mothers that calved female calves produced more milk and more fat in the first lactation. The first subsequent pregnancy with a female fetus also increased these yields. With these results, we confirmed the results published by Hinde and colleagues [1]. Their research also predicted that milk synthesis in the first lactation was affected not only by the sex of a born calf but also by the sex of the fetus carried by the mother during the pregnancy during lactation. They observed that the programming of the mammary gland as a reaction to the sex of the fetus continues in the next lactation, considering that the ability of milk synthesis was cumulative. This research [1] covered 1.49 million Holstein cows and analyzed 2.39 million lactation production records between 1995 and 1999. Cows that calved daughters (female calves) produced, on average, 142 ± 65.4 kg more milk in the first standard lactation. An increase in milk yield in the first two lactations of 2.7% (445 kg), if the first two calves are females (HH), is the highest recorded to date. This research further shows that sex did not affect the content of fat and protein, i.e., the content of milk. In papers by several other authors, it was established that the fat content varies depending on sex [11], breed [27,28], as well as lactation stages and lactation order [29].

Additionally, in several other studies [11,26,30,31], mothers of female offspring also had higher milk yield, but these differences compared to the male sex differed depending on lactation order or cattle breed. Studies conducted by some of the researchers [10] also indicate slightly higher milk production in mother cows that calved female calves during four consecutive lactations. These authors state that the probable cause of this phenomenon is the higher frequency of difficult calvings that occur in cows at the birth of a male calves, as a rule, have a higher body weight than female calves. Similar to a previously mentioned study [10], research preformed in New Zealand [11] established a small, but positive effect of the female sex on the overall milk yield of Holstein Friesian and Jersey cows. The positive effect of female calves was associated with higher milk yield only in the second lactation of Holstein Friesian cows (0.24%; *p* = 0.01) and higher milk yield in the third lactation of Jersey cows (1.1%; *p* = 0.01). Cows that calved male calves have a prolonged gestation period by, on average, 2 days, and thus a shorter lactation period, so the addition of a covariable for lactation duration in the animal model results show that Holstein Friesian cows that calve female calves have a higher milk yield only in the second lactation.

On the other hand, other authors [12,13,14] established that mothers achieve higher milk yields in the case of the birth of male rather than female offspring. [14] Research conducted by some of the authors [14] reported that cows produce 0.28% more milk in the first lactation if they calve a male calf compared to a female calf. The achieved difference was even greater when cows calved a second male calf, so that having two male calves results in a difference of 0.52% in milk production compared to any other combination of sexes of the offspring.

Some of the researchers’ results [32] support the hypothesis that milk production in high-producing cows may result in the birth of calves with lower body weight. It is assumed that carrying a larger calf during pregnancy causes a higher distribution of nutrients to the fetus, thus reducing milk production. In this regard, the birth of male calves could reduce milk production in the next lactation, also due to the increased number of assisted calvings [33,34]. In contrast to all mentioned studies, some of the authors [35], state that the sex of a Holstein cow calf had no effect on milk yield in a lactation of 305 days. A similar conclusion was reached by Afzal and colleagues [36] who established that calf sex did not affect milk yield in the buffalo. Their study established a statistically significant (*p* < 0.01) effect of sex on milk yield in the first three lactations. The differences in the significance of sex in the above studies on maternal milk yield could partly be explained by the differences in the used data on milk yield, duration of lactation and the applied models.

Some of the recent studies dealing with the issue of biased investment by mother mammals depending on the sex of the offspring do not fully support the TW hypothesis. For example, when it comes to mammalian milk yield, [7] places special emphasis on factors, such as quality and genetic predisposition of both parents, mother’s environment in early life, constitution, earlier puberty and maternal reproductive ability, maternal effect, etc. Today, there is more compelling evidence that suggests that the investment in offspring depends on both the father and the mother [7,37]. According to some authors [14], further investigations should be conducted in order to address the issue of conditions under which cattle populations are reared. It would also be important to test the TW hypothesis in view of investments in cow milk production harmonized with animal welfare.

We were the first to investigate the effect of the sex of calves at first and second calving on milk yield and fat yield in the first and second standard lactation, depending on milk-production levels on farms.

Having in mind that research is showing that the sex of calves affects milk yield traits, this could be used for the purpose of the wider utilization of sexed semen in dairy herds. Sexed semen is now widely available to milk producers who primarily use it to increase the number of female calves and heifers with high genetic potential [16]. According to some authors [38], milk yields were similar in heifers inseminated with sexed and conventional semen with the finding that the overall economic gain was higher for heifers obtained from sexed than from conventional semen. Thus, the use of sexed semen for the first insemination of heifers reduces the cost per female calf, has a positive effect on milk yield and allows for a quick return on investment.

## 5. Conclusions

Examining the biased effect of the sex of calves on the expression and phenotypic variability of milk yield traits, we established that the highest milk and fat yield was achieved in cows that calved female calves in the first two calvings (HH). The obtained results on the positive effect of female calves on milk production in the first and second lactation could be successfully applied and combined with greater use of sexed semen in dairy cattle, under these conditions. Further research on this topic should also include data on calf body weight, calving ease, duration of pregnancy, as well as on the effect of management, which is extremely important for the level of milk production on dairy farms.

## Figures and Tables

**Table 1:** Selection criteria for different categories.

Categories	Criterion
Bulls for insemination	Only Holstein bulls Semen from the same bulls was used on all analyzed farms Data (dates) of all inseminations during the period from 1982 to 2017 are known
Cows	Holstein type cows, and the average share of Holstein Friesian genes for animals covered by the analysis was 84%. Both parents, i.e., the origins are known No sexed semen or recombinant bovine somatotropin were used for cows covered by the analysis All cows remain in the same herd-farm from the first to the last calving Data about the sex of calves are known for every calving
Farms	Farms were divided into two groups (above average and below average) based on two criteria Average milk yield in the first two standard lactations was 14,337, i.e., 6738 kg for the first and 7599 kg for the second lactation. Body weight for two categories (first calvers and cows) First calvers—below average farms < 520 kg First calvers—above average farms > 520 kg Cows—below average farms < 670 kg Cows—above average farms > 670 kg

**Table 2:** Milk yield ($\bar{X}$ ± SD ) in a standard 305-day lactation (kg) depending on the combination of the sex of offspring after two consecutive calvings and production levels by farms in the first two lactations.

Combinations of Calf Sexes at First and Second Calving	Farms with Below Average Milk Production in the First Two Lactations	Farms with Above Average Milk Production in the First Two Lactations
	MY, kg $\bar{X}$ ± SD	MY, kg $\bar{X}$ ± SD
HH	13,027 ± 2794 (*n* = 1083)	14,750 ± 3608 (*n* = 2735)
HB	12,914 ± 2692 (*n* = 1169)	14,691 ± 2993 (*n* = 2817)
BH	13,130 ± 2805 (*n* = 1117)	14,525 ± 3001 (*n* = 2480)
BB	12,844 ± 2662 (*n* = 1158)	14,587 ± 3080 (*n* = 2622)

$\bar{X}$ —mean; SD—standard deviation; MY—milk yield; The values between observed groups (HH, HB, BH, BB) are statistically significant. (*p* < 0.01).

**Table 3:** Effect of calf sex on milk yield ($\bar{X}$ ± SD) and fat yield ($\bar{X}$ ± SD) in the first two standard lactations.

Item	1st Lactation		2nd Lactation	
Sex	H	B	H	B
(*n*)	(*n* = 7830)	(*n* = 7351)	(*n* = 7362)	(*n* = 7819)
MY, kg ($\bar{X}$ ± SD)	6777 ^A^ ± 1564	6697 ^B^ ± 1563	7601 ^C^ ± 1902	7597 ^D^ ± 1888
FY, kg ($\bar{X}$ ± SD)	242 ^a^ ± 53.74	240 ^b^ ± 54.68	270 ± 65.45	269 ± 65.32

$\bar{X}$ —mean; SD—standard deviation; MY—milk yield; FY—fat yield; B—bull; H—heifer; Means with the different upper capital letters differ significantly at *p* ≤ 0.01; Means with the different upper small letters differ significantly at *p* ≤ 0.05.

**Table 4:** Statistical significance of effects (F, estimate ± SE and *p* values) on milk yield (MY) and fat yield (FY).

Trait/Source	DF	MY, kg		FY, kg	
		F-Value Estimate ±SE	*p*-Value	F-Value Estimate ±SE	*p*-Value
Farm	6	368 *** −69.2 ± 3.6	<0.001	516 *** −4 ± 0.2	<0.001
Year of first calving	31	382 *** 117.8 ± 1.9	<0.001	203 *** 6 ± 0.4	<0.001
Season of calving	3	0.12 ^ns^ −1.9 ± 6.6	=0.8612	0.10 ^ns^ −0.09 ± 0.1	=0.912
Lactation order	1	412 *** 869.4 ± 14.6	<0.001	270 *** 7 ± 0.3	<0.001
Share of genes of the HF breed	4	205 *** 148.2 ± 9.9	<0.001	235 *** 5 ± 0.1	<0.001
Sex (H,B)	1	51 ** 15.6 ± 14.5	<0.01	29 ** 2 ± 0.6	<0.01
Level of milk yield on farms (above and below-average milk production)	1	285 *** 248 ± 15	<0.001	8.4 * 1 ± 1.2	<0.05
Combination of calf sexes at first and second calving	3	33 ** 15.6 ± 14.5	<0.01	0.85 ^ns^ −0.9 ± 5	=0.771
Interaction between sex and lactation order	3	47 ** 16.3 ± 15.1	<0.01	0.91 ^ns^ 1.1 ± 7	=0.861
Interaction between the combination of calf sexes (sex class) and level of milk yield on farms	7	67 ** 13.6 ± 12.6	<0.01	9.6 * 1.2 ± 1.5	<0.05

SE—standard error; DF—degree of freedom; MY—milk yield; FY—fat yield; *p* < 0.001 ***; *p* < 0.01 **; *p* < 0.05 *; *p* > 0.05 ^ns^; ns—non significant.

**Table 5:** Effect of the combination of sex of calves at the first and second calving on milk yield ($\bar{X}$ ± SD) and fat yield ($\bar{X}$ ± SD) in the first and second standard lactation.

Combinations of Calf Sexes at First and Second Calving	MY, kg		FY, kg	
	1st Lactation $\bar{X}$ ± SD	2nd Lactation $\bar{X}$ ± SD	1st Lactation $\bar{X}$ ± SD	2nd Lactation $\bar{X}$ ± SD
HH	6801 ^A^ ± 1562 (*n* = 3788)	7634 ^E^ ± 1909 (*n* = 3788)	243 ^A,a^ ± 53.74 (*n* = 3788)	270 ^B^ ± 65.45 (*n* = 3788)
HB	6754 ^B^ ± 1566 (*n* = 3563)	7621 ^F^ ± 1892 (*n* = 3563)	241 ^A^ ± 54.20 (*n* = 3563)	269 ^B^ ± 65.32 (*n* = 3563)
BH	6685 ^C^ ± 1558 (*n* = 4031)	7563 ^G^ ± 1894 (*n* = 4031)	239 ^A,b^ ± 54.50 (*n* = 4031)	268 ^B^ ± 65.07 (*n* = 4031)
BB	6709 ^D^ ± 1569 (*n* = 3799)	7572 ^H^ ± 1884 (*n* = 3799)	240 ^A^ ± 54.68 (*n* = 3799)	268 ^B^ ± 65.53 (*n* = 3799)

$\bar{X}$ —mean; SD—standard deviation; MY—milk yield; FY— fat yield; means with the different upper capital letters (A, B, C, D, E, F, H) differ significantly at *p* ≤ 0.01; means with the different upper small letters (a, b) differ significantly at *p* ≤ 0.05.

**Table 6:** Effect of the combination of sex of calves during the first and second calving on milk yield ($\bar{X}$ ± SD) and fat yield ($\bar{X}$ ± SD) in the 1st and 2nd standard lactation depending on the level of production on farms.

Combinations of Calf Sexes at First and Second Calving	Farms with Below Average Milk Production		Farms with Above Average Milk Production		Farms with Below Average Milk Production		Farms with Above Average Milk Production	
	MY, kg $\bar{X}$ ± SD		MY, kg $\bar{X}$ ± SD		FY, kg $\bar{X}$ ± SD		FY, kg $\bar{X}$ ± SD	
	1st Lactation	2nd Lactation	1st Lactation	2nd Lactation	1st Lactation	2nd Lactation	1st Lactation	2nd Lactation
HH	6150 ^A,B,¶^ ± 1346	6877 ^C,§^ ± 1781	6962 ^A,£^ ± 1529	7788 ^C,¥^ ± 1879	224 ^£^ ± 48.41	248 ^A,B,C,¶^ ± 64.06	246 ^¶^ ± 50.76	271 ^§^ ± 61.44
HB	6093 ^A,¶^ ± 1366	6821 ^D,§^ ± 1671	6928 ^B,£^ ± 1511	7762 ^C,D,¥^ ± 1830	221 ^£^ ± 48.87	245 ^A^ ± 50.28	245 ^¶^ ± 54.77	271 ^§^ ± 61.73
BH	6161 ^A,B,¶^ ± 1391	6868 ^E,§^ ± 1692	6820 ^A,B,£^ ± 1501	7705 ^D,¥^ ± 1846	223 ^£^ ± 49.32	251 ^B,¶^ ± 62.04	242 ^D,¶^ ± 50.45	269 ^§^ ± 60.96
BB	6061 ^B,¶^ ± 1327	6783 ^F,§^ ± 1670	6821 ^A,B,£^ ± 1488	7774 ^C,D,¥^ ± 1886	220 ^£^ ± 46.76	243 ^C,¶^ ± 58.61	243 ^D,¶^ ± 53.32	270 ^§^ ± 62.94

$\bar{X}$—mean; SD—standard deviation;MY—milk yield; FY—fat yield; (A,B,C,D)—calf sex combinations (HH, HB, BH, BB, respectively); (¶, §)—lactation (first and second, respectively); (£, ¥)—production levels (two levels—above and below average, respectively); Means with the different subscripts (A, B, C, D, ¶, §, £, ¥) differ significantly at *p* ≤ 0.01.

## Data Availability

The data used in this study are available on request from the corresponding author.
